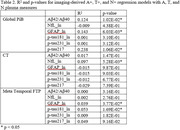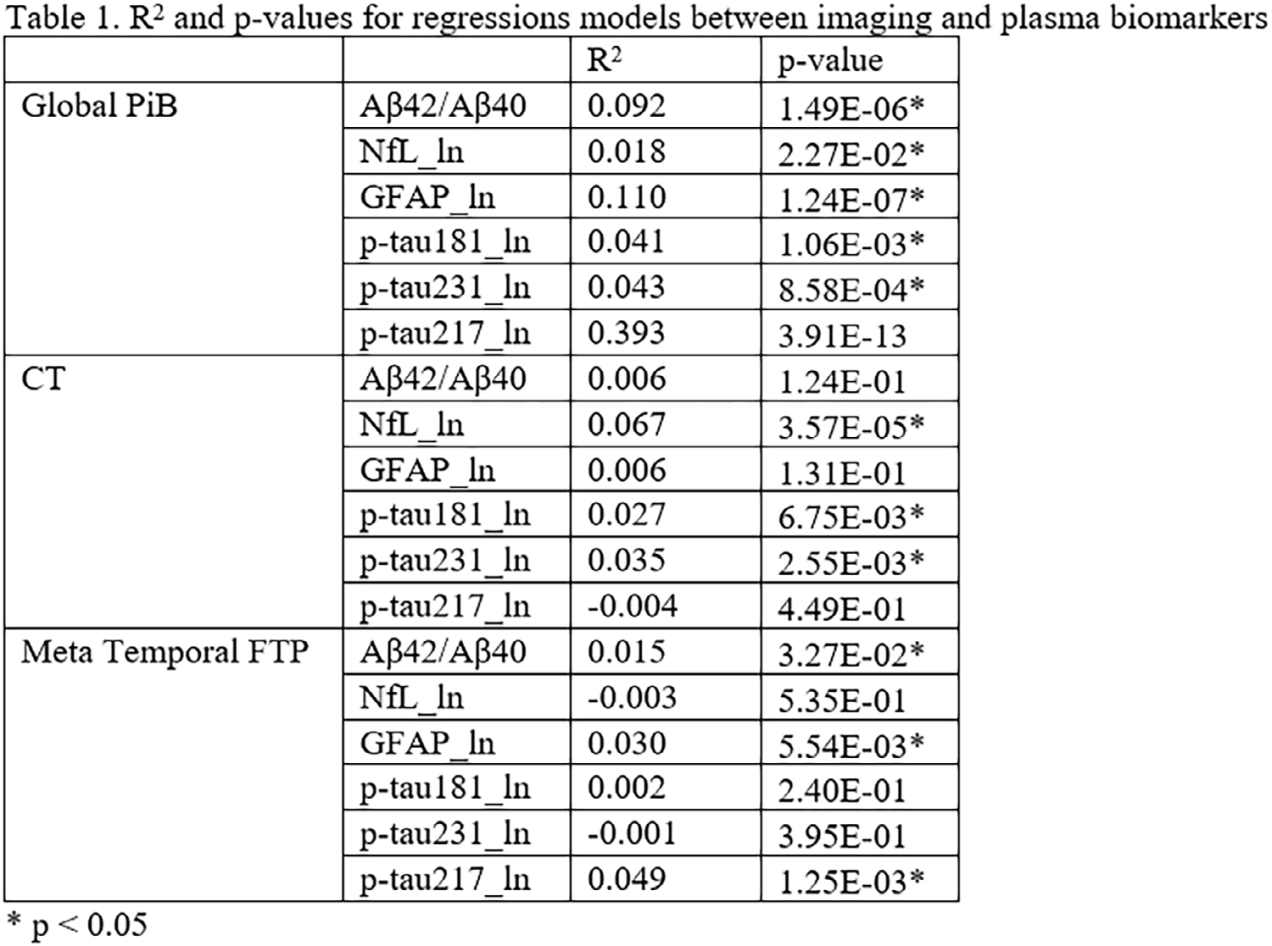# Assessing the correlations between imaging and plasma biomarkers within the AT(N) framework

**DOI:** 10.1002/alz.093085

**Published:** 2025-01-09

**Authors:** Alexandra Gogola, Ann D Cohen, Xuemei Zeng, Brian J Lopresti, Beth E. Snitz, Dana Tudorascu, Davneet S Minhas, Milos D Ikonomovic, Tharick A. Pascoal, Julia Kofler, Cristy Matan, Neale S Mason, Howard J Aizenstein, Chester Mathis, William E Klunk, Henrik Zetterberg, Kaj Blennow, Oscar L. Lopez, Victor L Villemagne, Thomas K Karikari

**Affiliations:** ^1^ University of Pittsburgh, Pittsburgh, PA USA; ^2^ University of Gothenburg, Mölndal, Gothenburg Sweden; ^3^ University of Gothenburg, Gothenburg Sweden; ^4^ University of Gothenburg, Mölndal Sweden

## Abstract

**Background:**

Given the increasing usage of plasma biomarkers for Alzheimer’s disease (AD) studies, it is necessary to better understand relationships between plasma biomarker and PET and MR imaging outcomes, particularly within the AT(N) framework.

**Method:**

We evaluated plasma samples from 233 subjects (age 74.05.9y) who underwent 3T MR and both [^11^C]PiB and [^18^F]flortaucipir (FTP) PET imaging. SUVR values were calculated for Global PiB, and Meta‐Temporal FTP. An AD signature cortical thickness (CT) composite was derived from MR images using FreeSurfer 5.3, Blood samples were processed and sampled to obtain measures of plasma Aβ42, Aβ40, p‐tau181, p‐tau217, p‐tau231 and neurofilament light (NfL), and glial fibrillary acidic protein (GFAP). Imaging outcomes were split into infra (‐) and supra (+) threshold groups using 1.35 SUVR for Global PiB, 1.18 SUVR for Meta‐Temporal FTP, and 2.7 mm for CT. Plasma Aβ was expressed as Aβ42/Aβ40 ratio while p‐tau181, p‐tau217, p‐tau231, NfL, and GFAP were natural‐log‐transformed to normally distribute the data. Simple regression models between continuous variables of each imaging‐derived measure and all plasma biomarkers were evaluated.

**Result:**

Full group regression model R^2^ and regional p‐values are shown in Table 1, suprathreshold regressions are shown in Table 2. Full Global PiB was significantly associated with all plasma biomarkers while CT was associated with NfL, p‐tau181, and p‐tau231. Meta‐Temporal FTP was associated with GFAP and p‐tau217. Additionally, A+Global PiB was significantly associated with Aβ42/Aβ40, GFAP, and p‐tau217; (N+) CT with NfL; and T+ Meta‐Temporal FTP with p‐tau181 and GFAP.

**Conclusion:**

The significant associations between suprathreshold imaging and plasma biomarkers suggests that, in A+, T+, or (N+) both capture abnormal AD pathophysiology. Further, the absence of an association in the infrathrehold cases supports that the suprathreshold associations are not artifactual. Future work will explore plasma thresholds and concordance between imaging and plasma AT(N).